# In ovo administration of a phage cocktail partially prevents colibacillosis in chicks

**DOI:** 10.1016/j.psj.2023.102967

**Published:** 2023-07-26

**Authors:** Marianne Nicolas, Arnaud Faurie, Mylène Girault, Sébastien Lavillatte, Pierrette Menanteau, Thierry Chaumeil, Mickael Riou, Philippe Velge, Catherine Schouler

**Affiliations:** ⁎ INRAE, University of Tours, ISP, F-37380 Nouzilly, France; † INRAE, UE PFIE, F-37380 Nouzilly, France

**Keywords:** avian colibacillosis, prevention, phage cocktail, in ovo

## Abstract

Avian pathogenic *Escherichia coli* (**APEC**) causes colibacillosis, the main bacterial disease in poultry leading to significant economic losses worldwide. Antibiotic treatments favor the emergence of multidrug-resistant bacteria, and preventive measures are insufficient to control the disease. There is increasing interest in using the potential of bacteriophages, not only for phage therapy but also for prevention and biocontrol. This study aimed to evaluate the efficacy of a phage cocktail administered in ovo to prevent avian colibacillosis in chicks. When 4 different phages (REC, ESCO3, ESCO47, and ESCO58), stable under avian physiological conditions, were combined and inoculated at 17 embryogenic days (**ED**), they were transmitted to the newly hatched chicks. In a second trial, the 4-phage cocktail was inoculated into the allantoic fluid at ED16 and after hatch 1-day-old chicks were challenged with the O2 APEC strain BEN4358 inoculated subcutaneously. Two phages (REC and ESCO3) were still detected in the ceca of surviving chicks at the end of the experiment (7-days postinfection). Chicks that received the phages in ovo did not develop colibacillosis lesions and showed a significant decrease in intestinal BEN4358 load (8.00 × 10^7^ CFU/g) compared to the challenged chicks (4.52 × 10^8^ CFU/g). The majority of the reisolated bacteria from the ceca of surviving chicks had developed full resistance to ESCO3 phage, and only 3 were resistant to REC phage. The partially or complete resistance of REC phage induced a considerable cost to bacterial virulence. Here, we showed that phages inoculated in ovo can partially prevent colibacillosis in 1-wk-old chicks. The reduction in the APEC load in the gut and the decreased virulence of some resistant isolates could also contribute to control the disease.

## INTRODUCTION

Avian colibacillosis is the main bacterial disease of poultry, affecting all birds in all types of poultry production at all ages. Colibacillosis is induced by avian pathogenic *Escherichia coli* (**APEC**) strains, which colonize the chicken intestine asymptomatically. APEC cause a large variety of extraintestinal syndromes that vary according to the age of the animal. During the first week after hatching, chicks are highly susceptible to *E. coli* infections. Omphalitis and yolk sac infection occurs through fecal contamination of eggs or in ovo during egg formation when laying hens suffer salpingitis/peritonitis/salpingoperitonitis syndrome. During these infections, APEC strains can disseminate into the bloodstream, inducing septicemia, and affect extraintestinal organs, inducing perihepatitis, pericarditis, and airsacculitis (Nolan et al., 2020).

The emergence of multidrug-resistant bacteria, coupled with the transfer of antibiotic resistance genes between animal and human bacterial strains, led to the withdrawal of the use of antibiotics as growth promoters in Europe in 2006 (Castanon, 2007). Therefore, prevention of avian colibacillosis is primarily achieved through biosecurity measures (e.g., disinfection of eggs after laying and elimination of cracked and soiled eggs) and by vaccination (Nolan et al., 2020). APEC strains are highly diverse and many serogroups circulate (Schouler et al., 2012). Vaccines that are currently marketed do not protect birds against all APEC strains, targeting only a few serogroups (Kariyawasam et al., 2004; Abd El-Mawgoud et al., 2020). Therefore, new preventive strategies are needed to decrease the impact of APEC strains, one such approach is phage therapy.

Several reports have demonstrated the success of phage therapy in the treatment of colibacillosis in experimentally infected chickens, with different application routes. Barrow et al. demonstrated complete protection when phage R was administered simultaneously with the challenge strain, either intramuscularly or intracranially (Barrow et al., 1998). Mortality was significantly decreased from 57 to 13% in the birds given a single intramuscular injection of a 2-phage cocktail immediately after *E. coli* challenge (Huff et al., 2003b). However, these routes and timing of phage administration are not practical for field conditions. Administration of bacteriophages per os in drinking water or by aerosolization have proved to be ineffective in treating or preventing infection or reducing clinical symptoms (Huff et al., 2002a,b; 2003b, 2013; Tsonos et al., 2014; Kittler et al., 2020).

In ovo injection of vaccines has commonly been used since 1980, and has proven to protect birds against many diseases (Marek's disease, infectious bursal disease or coccidiosis) (Peebles, 2018). It has also been shown that birds could be protected from an *E. coli* infection by in ovo administration of probiotic or antimicrobial (Arreguin-Nava et al., 2019; Nguyen et al., 2021). Therefore, in ovo administration of a phage cocktail could be a promising means to prevent chick mortality or morbidity.

In a previous study, we isolated and characterized 19 coliphages belonging to 9 taxonomic genera (Nicolas et al., 2023). An 8-phage cocktail was able to fully inhibit the multiplication of the APEC BEN4358 strain in vitro. Moreover, in a chicken embryo lethality assay, the cocktail allowed 90% of treated embryos to survive the bacterial infection, compared to 0% of nontreated embryos. The present study aimed to test whether in ovo administration of a 4-phage cocktail composed of the phages REC, ESCO3, ESCO47, and ESCO58 could protect chicks challenged with the APEC BEN4358 strain on their first day of life.

## MATERIALS AND METHODS

### Bacterial Strain and Culture Media

BEN4358, an O2:K1 NalR APEC strain, isolated in 2013 in France from a layer chicken suffering from colibacillosis, was used in this study. The strain was grown on Tryptic Soy Agar (**TSA**) plates or in Lysogeny Broth (**LB**, Miller formulation) medium overnight (18 h) at 37°C with shaking (180 rpm) and stored at −80°C in 15% (v/v) glycerol (Sigma-Aldrich, St-Louis, MO).

### Phages Used and Amplification

Four phages belonging to the class *Caudoviricetes* (*Phapecoctavirus* REC, *Nonagvirus* ESCO3, *Mosigvirus* ESCO47, and *Tequatrovirus* ESCO58) were previously isolated and characterized (Nicolas et al., 2023). Phages were multiplied in BEN4358 strain at logarithmic growth phase (OD_600 nm_ ∼0.4) in 5 mL of LB supplemented with MgSO_4_ (10 mM) (Sigma-Aldrich) and CaCl_2_ (1 mM) (Sigma-Aldrich), with the phage at a multiplicity of infection (**MOI**) = 0.1. After 15 min at room temperature without shaking to allow phage adsorption, the culture was incubated for 4 h at 37°C with shaking (180 rpm). The culture was centrifuged at 3,800 × *g* (Beckman, GS-15R Centrifuge), and the supernatant was filtered through 0.2 µm pore-size microfilters (ClearLine) to remove bacterial debris. Lysates were stored at 4°C. The presence of phages was confirmed by spot test assays (Kutter, 2009).

### Phage Stability

Stability of the phages was analyzed at 37°C, the embryo incubation temperature, and 41°C, the body temperature of healthy chicken (Prinzinger et al., 1991). Approximately 10^5^ PFU/mL of phages were suspended in Dulbecco's phosphate-buffered saline (**DPBS**, Sigma-Aldrich) adjusted with MgSO_4_ (10 mM) and CaCl_2_ (1 mM). Phage titer was determined at 30 min, 60 min, 24 h, and 48 h of incubation at 37°C or 41°C, through 10-fold serial dilution spot tests in DPBS on a BEN4358 bacterial lawn, as described above. In addition, stability of the phages in simulated intestinal fluid (**SIF**) was determined as described by Colom et al. (2017). Approximately 10^5^ PFU/mL of phages were suspended in SIF medium (DPBS, 10 mM MgSO_4_, 1 mM CaCl_2_, 1 mg/mL pancreatin (Sigma-Aldrich), 0.3% bile salts (Sigma-Aldrich), 0.85% NaCl (Sigma-Aldrich), pH 8). Each phage was titrated at 0, 30, 60, and 120 min of incubation at 41°C with agitation (90 rpm) by 10-fold serial dilution spot tests in DPBS on a BEN4358 bacterial lawn. These experiments were performed 3 times for each phage.

### Ethical Statement

All animal experiments were carried out according to EU directives and French regulations (Directive 2010/63/EU, 2010; Rural Code, 2018; Décret no. 2013-118, 2013), and were evaluated by the ethics committee of Centre Val de Loire (CEEA VdL no. 19) and approved by the Ministry of Higher Education and Research (APAFIS #26717-2020072413185773 v4).

### Animal Trials

Two trials were performed at the Infectiology of Farm, Model and Wildlife Animals Facility (PFIE, Centre INRAE Val De Loire: https://doi.org/10.15454/1.5572352821559333E12; member of the National Infrastructure EMERG'IN) using specific-pathogen-free embryonated eggs (White Leghorn, PA-12, France). The incubation and inoculation conditions were performed in accordance with Trotereau et al. (Trotereau and Schouler, 2019), except for the embryonic days (**ED**) of inoculation (ED16 or ED17). The eggs were placed in an egg flat with the air-cell up. The edge of the air-cell was marked. After disinfecting the marked area, a small hole was drilled using an 18G (1.2 × 40 mm) sterile needle. A 1 mL syringe with a 25G (0.5 × 16 mm) needle was used to inoculate eggs. The needle was inserted to the hub while holding the syringe vertically and 100 µL of phage cocktail solution was injected into the allantoic cavity. At ED19, the embryonated eggs were transferred into isolators for hatching.

***First Trial.*** This trial was performed to assess whether phages inoculated in ovo were maintained in newly hatched chicks. The phage cocktail consisted of 25% of the lysates of the phages REC, ESCO3, ESCO47, and ESCO58. The final concentration of the phage cocktail was 4.3 × 10^7^ PFU/mL. At ED17, 100 µL of the phage cocktail were inoculated in 25 embryonated eggs. All the newly hatched chicks were sacrificed and necropsied to collect the yolk sac and the ceca to monitor the presence of phages. Collected organs were placed in Miltenyi tubes previously filled with 3.5 mL of physiological water and weighed, then homogenized for 1 min by the gentleMACS dissociator (Miltenyi Biotec, Paris, France).

***Second Trial.*** This trial was performed to assess the protective potential of the 4-phage cocktail inoculated in ovo into chicks challenged with an APEC strain at 1-day old. Eggs were inoculated at ED16 with either 100 µL of the phage cocktail (5.2 × 10^8^ PFU/mL) or DPBS. Then, at 1-day posthatch, the chicks were challenged by subcutaneous inoculation in the breast region under the right wing with either 200 µL of BEN4358 strain (2.5 × 10^3^ CFU) or DPBS. The bacterial inoculum was prepared as follow: an overnight culture in LB was diluted in DPBS to approximately obtain 10^4^ CFU/mL and was enumerated on TSA plates. One group (“Phages”) had received the 4-phage cocktail in ovo, and DPBS was injected the day after hatching (*n* = 10). The second group (“EC”) had received DPBS in ovo and was challenged with BEN4358 strain (*n* = 17). The third group (“EC + Phages”) had received the 4-phage cocktail in ovo and was challenged with the APEC strain (*n* = 18).

Following the challenge, the chicks were placed in the isolator in which they were born. The air in the enclosure was filtered at intake and exhausted by H14 filters to protect the animals and the environment. The temperature was adapted to the age of the animals and controlled by a probe inside the enclosure. The chicks had a surface area of 1 m² at their disposal, on a grid adapted to their age (mesh size 0.5 cm × 0.5 cm in this case). The environment was enriched by the addition of racks, carpeted areas and hanging objects, and social enrichment by group. Water and antibiotic-free feed were provided ad libitum. All animals were identified by a metal ring bearing a unique number. Health status of the chicks was monitored twice-daily by the animal keepers, looking for: visible respiratory difficulty; prostration; ruffled feathers; no response to stimulation; no feeding. Animals were euthanized if their clinical condition left no doubt that they were suffering. In particular, the association of at least 3 major clinical signs such as: ruffled feathers, prostration, anorexia, complaint... for a maximum of 24 h, led to the euthanasia of the animals. Animals reaching the experimental limit point were euthanized by cervical elongation with forceps adapted to their weight (less than 250 g), that is, less than 15 d depending on the avian line. The mortality rate was recorded daily.

Seven-days postinfection (**dpi**), all surviving chicks were euthanized by cervical dislocation and necropsied. Macroscopic fibrinous lesions of the liver, left abdominal, posterior and anterior thoracic air sac, left lung, and pericardium were observed and scored as previously described (Brée et al., 1989; Alber et al., 2019) (Table 1). Liver (distal part of the left lobe) and one of the ceca were collected in 3.5 mL of physiological water Miltenyi tubes. The samples were weighed and homogenized as described above.

**Table 1:** Scoring of colibacillosis lesions.

Organ	Score	Lesions
Liver	0	No lesions
	1	Decolorization of lobes/small quantity of fibrinous exudate
	2	Large quantity of fibrinous exudate
Air sac	0	No lesions
	1	Slight opacity of membrane/medium quantity of fibrinous exudate
	2	Opacity of membrane/large quantity of fibrinous exudate
Lung	0	No lesions
	1	Slight opacity/small quantity of fibrinous exudate
	2	Marked opacity/large quantity of fibrinous exudate
Heart	0	No lesions
	1	Opacity/vascularization/small quantity of fibrinous exudate
	2	Large quantity of fibrinous exudate

### Bacterial Enumeration and Identification From Recovered Organs

Ten-fold serial dilutions of 1 mL homogenized samples of liver and ceca were plated on Drigalski plates (Bio-Rad Laboratories Inc., Hercules, CA) supplemented or not with nalidixic acid (30 mg/mL, Sigma) for bacterial quantification.

One colony of each sample was tested using PCR-based serotyping to confirm that the clones belonged to the same serogroup as the BEN4358 strain. A part of the colony was taken with a toothpick and placed in 13.3 µL of water. A BEN4358 colony was used as a control. A PCR targeting the O2 antigen, with the primers gndbis.f (5′-ATACCGACGACGCCGATCTG-3′) and rfbO2a.r (5′-GTGACTATTTCGTTACAAGC-3′) (Clermont et al., 2007), was used to amplify the target gene. The PCR reaction was performed as follows: each mix of 25 µL final volume contained the 13.3 µL of resuspended-colony in water, 5 µL of 5× Green GoTaq reaction buffer (Promega, Madison, WI), 1.5 µL of MgCl_2_ (25 mM) (Promega), 0.5 µL of dNTP (10 µM) (Promega), 1.25 µL of each primer (10 µM) (Eurogentec, Liege, Belgium), and 0.2 µL of GoTaq DNA Polymerase (Promega). The thermocycling conditions were: 5 min at 94°C, 30 cycles of 30 s at 94°C, 30 s at 55°C, and 30 s at 72°C, and a final step of 5 min at 72°C (ProFlex, Applied Biosystems, Waltham, MA). PCR products were separated by electrophoresis in 1% agarose (Eurogentec) gels in 0.5× tris-acetic acid-EDTA (**TAE**) buffer (Sigma-Aldrich) with 1/500 Midori Green Advance (Dutscher, Batley West Yorkshiren UK), and visualized under UV using GelDoc Go Imaging System (Bio-Rad Laboratories Inc.).

### Phage Enumeration and Identification From Recovered Organs

One milliliter of each homogenized liver and ceca sample was centrifuged for 10 min at 9,300 × *g* (Centrifuge 5415R, Eppendorf, Hamburg, Germany). The supernatant was filtered through a 0.2 µm multiscreen filter plate (Millipore, Billerica, MA) to remove bacterial cells. The presence of phages was detected using spot assays: 200 µL of BEN4358 strain in logarithmic growth phase (OD_600 nm_ ∼0.4) was added to 5 mL of LB agarose 0.5% (w/v) maintained at 55°C, CaCl_2_ (1 mM), MgSO_4_ (10 mM), and 30 µM 2,3,5-triphenyltetrazolium chloride (**TTZ**) (Sigma), and poured onto a 1.5% (w/v) LB agar plate. Ten microliters of 10-fold dilutions of organ sample supernatants were spotted onto the solidified bacterial lawn and incubated overnight at 37°C. Based on the recovered weight of the organ, the detection threshold was around 6.0 × 10^2^ PFU/g.

Spots obtained at the lowest dilution were collected and mixed in 100 µL of sterile water, heated for 10 min at 99°C, then left for 1 h 30 at room temperature for diffusion. The phages were then identified through PCR, as previously described (Nicolas et al., 2023).

### Phage Susceptibility Testing

Thirteen colonies were reisolated from the ceca of each surviving chick in the “EC + Phages” group to determine their phage susceptibility through efficiency of plating (**EOP**) analysis. Ten microliters of serial 10-fold dilutions of a lysate of each phage were spotted on bacterial lawns of these reisolated clones, and plates were incubated overnight at 37°C. The inoculated BEN4358 strain was used as a control to determine the relative EOP value. The values were defined as follows: sensitive when EOP ≥0.5, moderate when 0.01  ≤ EOP < 0.5, and resistant when 0.0001 < EOP < 0.01 (Petsong et al., 2019).

### Chicken Embryo Lethality Assay

In order to determine the virulence of the reisolated clones, a chicken embryo lethality assay was performed, based on a method described by Trotereau and Schouler (2019). The virulence of 4 reisolated clones from the ceca of “EC + Phages” chicks (chicks nos. 14, 16, 18, and 23) was tested on groups of ten 12-day-old embryonated-eggs. As a control, the wild-type BEN4358 strain was inoculated into 10 eggs. The eggs were candled daily for 6 d to monitor embryo mortality.

### Capsular and O Antigens Detection

A latex agglutination test for K1 antigen detection (Wellcogen *N. meningitidis* B/*E. coli* K1, Fisher, Walthman, MA) was performed on BEN4358 and the 4 clones recovered from the ceca (nos. 14, 16, 18, and 23), in accordance with the manufacturer's instruction. In the presence of the K1 antigen, the latex particles agglutinated, whereas in its absence the particles remained in a homogeneous suspension. The expression of the O2 antigen was determined using an agglutination test based on specific antibodies (O2 coagglutin reagents, Ceva Biovac, Beaucouzé, France). Similarly, the presence of the O antigen was validated by the formation of agglutinates.

### Statistical Analysis

The results were analyzed using GraphPad Prism for Windows version 6.07 (GraphPad Software, La Jolla, CA). The log-rank test was applied for survival curves, represented by Kaplan-Meir curves. Due to asymmetry in the distribution of the data, the nonparametric Mann-Whitney test was used. Significance was considered at *P* values <0.05.

## RESULTS

### The 4 Phages Were Stable In Vitro Under Avian Physiological Conditions and Were Detected in Newly Hatched Chicks After In Ovo Inoculation

**Figure 1:**
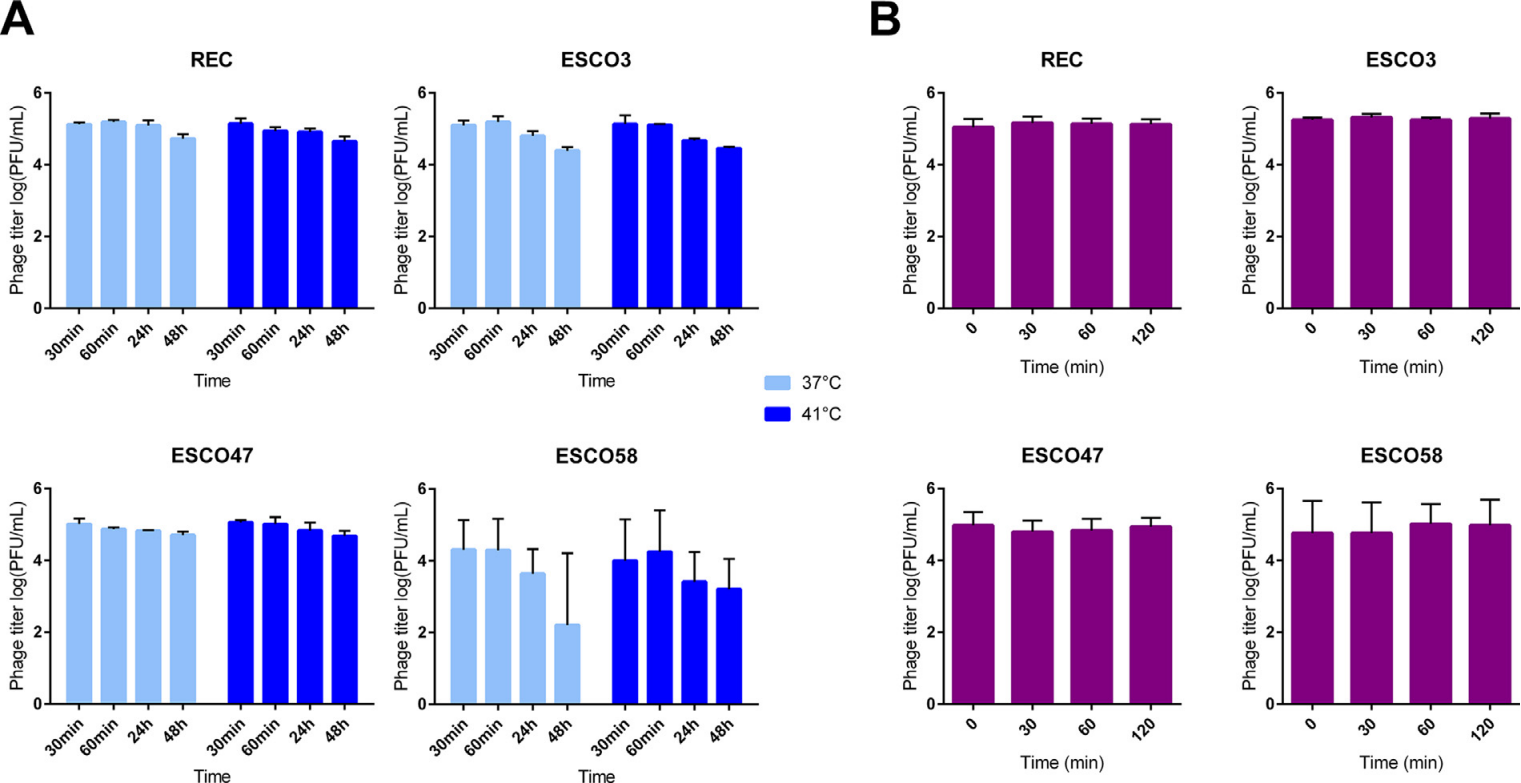
Stability study of the 4 phages. (A) Thermal stability of the phages was determined at 37°C and 41°C for 2 d by obtaining phage titer on BEN4358 strain by spot assay. (B) Phage stability in simulated intestinal fluid. Values represent mean ± SD of 3 independent experiments.

Phage stability was determined under egg incubation conditions and chick body temperature (37°C and 41°C, respectively). At 37°C, REC, ESCO3, and ESCO47 phages were relatively stable as only a slight decrease in phage titers was observed (0.39, 0.70, and 0.30 log units, respectively) (Figure 1A). However, the infectivity of ESCO58 was strongly reduced, a 2.1 log drop in phage titer was observed after 48 h of incubation (Figure 1A). The 4 phages showed a minor reduction in stability (<1 log unit) at 41°C, even for ESCO58 which was more stable at this temperature than at 37°C (Figure 1A). In addition, all 4 phages were stable in simulated intestinal fluid for up to 2 h (Figure 1B).

**Figure 2:**
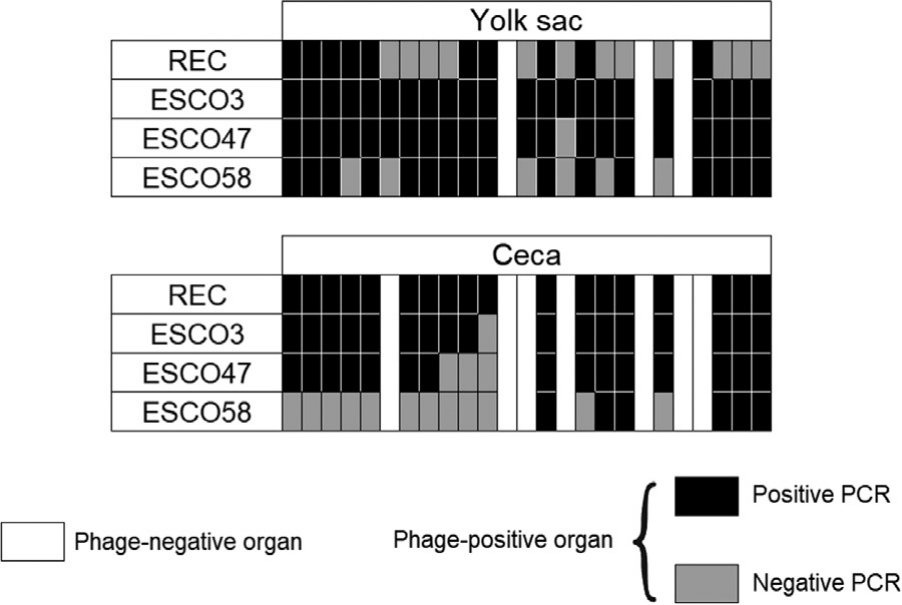
Presence of phages in the yolk sac and ceca of newly hatched chicks and identification of phages by PCR in phage-positive organs. Phage titer of yolk sac and ceca samples was determined using spot assays. Each column represents 1 chick (*n* = 25). The spots of the lower dilution of phage-positive organs were collected, mixed, and boiled in sterile water to perform PCR to identify the presence or absence of each individual phage. White boxes represent phage-negative organs, in which PCR identification was not performed; gray boxes represent negative PCR in phage-positive organs; black boxes represent positive PCR in phage-positive organs.

As the phages were relatively stable at 37°C, they were combined and the phage cocktail was inoculated in ovo at ED 17 to determine whether they could be transmitted to the newly hatched chick. The presence of phages was monitored in the yolk sac and in ceca of 25 newly hatched chicks. Phages were present in the yolk sac of 88% of newly hatched chicks at an average titer of 1.15 × 10^5^ PFU/g, and in the ceca of 72% of newly hatched chicks with an average titer of 7.48 × 10^4^ PFU/g. In phage-positive chicks, phage identification by PCR was performed: REC, ESCO3, ESCO47, and ESCO58 were detected in the yolk sac (45, 100, 95, and 72%, respectively) and in the ceca (82, 77, 68, and 28%, respectively) (Figure 2). Thus, the 4 phages were heterogeneously present in a majority of the newly hatched chicks. Surprisingly, 4 birds which were phage-positive in the yolk sac had no detectable phages in the ceca (Figure 2).

### Chicks That Received Phages at the Embryonic Stage Developed Less Colibacillosis Lesions and Had Less BEN4358 APEC Strain in the Intestine

**Figure 3:**
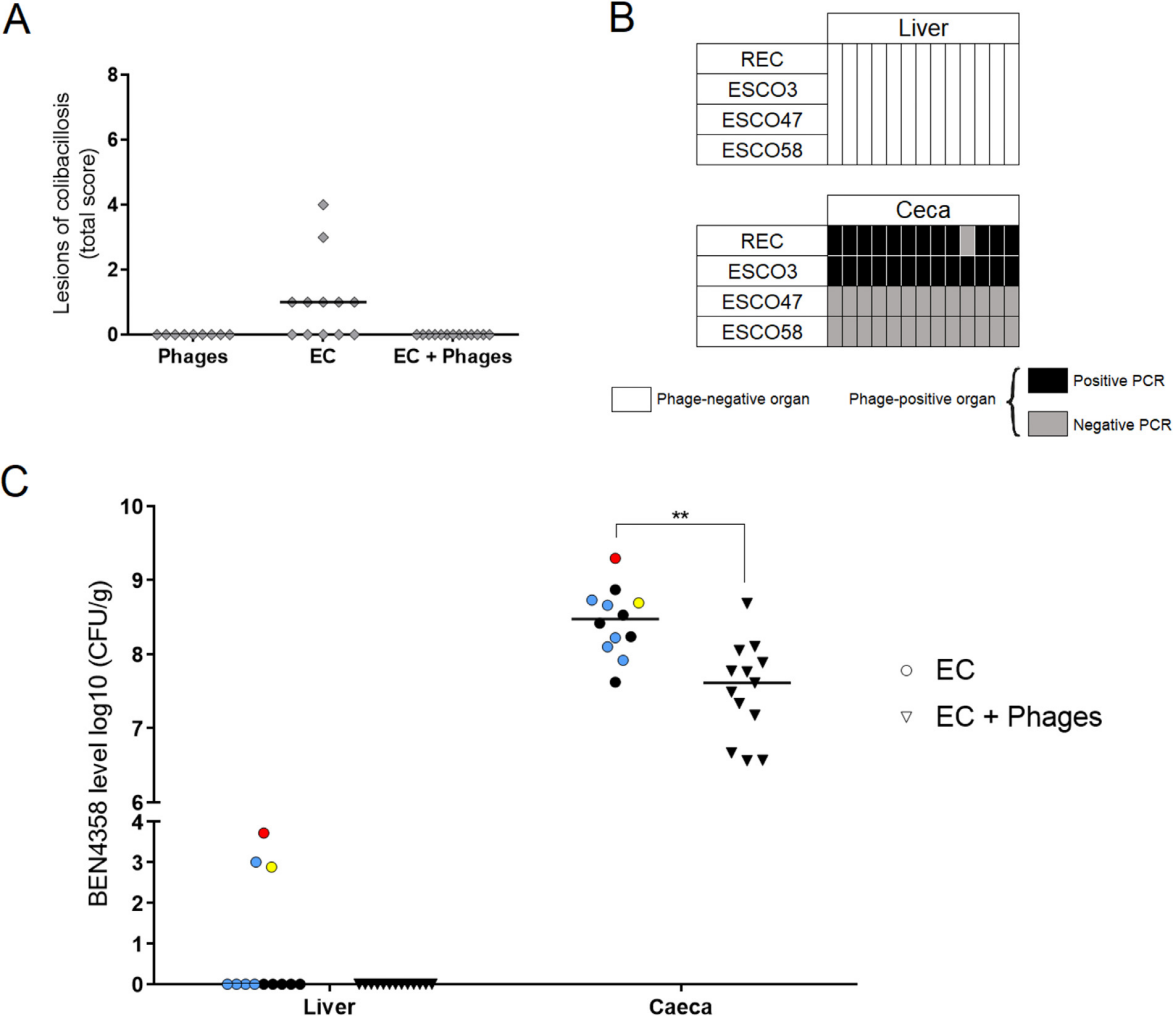
The 2 phages present in the ceca of surviving chicks at 7 dpi prevented the development of fibrinous lesions associated with colibacillosis and significantly reduced the intestinal load of BEN4358. (A) Colibacillosis lesions score. Each diamond represents 1 surviving chick. The lesion score was not determined for dead animals. Black bars indicate the median for each group. (B) Presence of phages in the liver and ceca of surviving chicks of the “EC + Phages” group and identification of phages by PCR in phage-positive organs. Phage titer in liver and ceca samples was determined by spot assays. Each column represents 1 chick (*n* = 13). The spots of the lower dilution of phage-positive organs were collected, mixed and boiled in sterile water to perform PCR to identify the presence or absence of each individual phage. White boxes represent phage-negative organs, in which PCR identification was not performed; gray boxes represent negative PCR in phage-positive organs; black boxes represent positive PCR in phage-positive organs. (C) Bacterial concentration in liver and ceca of surviving chicks of the “EC” (circle) and “EC + Phages” (triangle) groups. Each symbol represents 1 chick, and different colors were assigned according to the severity of lesions: black represents chicks with no lesions; blue represents chicks with liver lesions; yellow represents chicks with liver and pericardial lesions; and red represents chicks with liver, pericardial, and lung lesions. Data were analyzed using a Mann-Whitney test at a significance level of *P* < 0.05.

As the phages were transmitted to the newly hatched chicks, the cocktail's potential to protect 1-day-old chicks challenged with an APEC strain was assessed. Mortality rate at 7 dpi between the “EC” group (29.4%) and the “EC + Phages” group (27.8%) was not statistically different (*P* > 0.05). Typical fibrinous colibacillosis lesions were observed in 7 out of 12 surviving chicks of the EC group (Figure 3A). All 7 chicks had mild perihepatitis, and among them, 1 chick also had severe pericarditis, and another had moderate lung lesions and severe pericarditis. In contrast, no chicks that survived in the “EC + Phages” group showed colibacillosis lesions. This may suggest that the phages prevented the development of infection in the surviving chicks.

Phages were present in the ceca of the surviving chicks in the “EC + Phages” group, at an average concentration of 9.44 × 10^6^ PFU/g. The phage identification using PCR showed that ESCO3 was present in 13/13 chicks, REC in 12/13, while ESCO47 and ESCO58 were not detected (Figure 3B). No phages were detected in the unchallenged “Phages” group, which suggests that without multiplication of their bacterial host phages were gradually eliminated from the organism.

The bacterial load of BEN4358 was determined in the liver and ceca of surviving chicks. In the “EC” group, BEN4358 was present in the liver of 3 chicks that had fibrinous colibacillosis lesions, 2 of which had the highest lesion score (Figure 3C). In the ceca, the average BEN4358 load was significantly lower in the “EC + Phages” group than in the “EC” group (reduction of 0.9 log units) (*P* = 0.001) (Figure 3C). The chick with the most severe lesions had a higher bacterial load in the intestine than the other birds. PCR-based serotyping confirmed that the clones of each bacteria-positive organ were *E. coli* O2, like the wild-type strain (data not shown).

### Phage Resistance Might Have a Bacterial Virulence Cost

**Figure 4:**
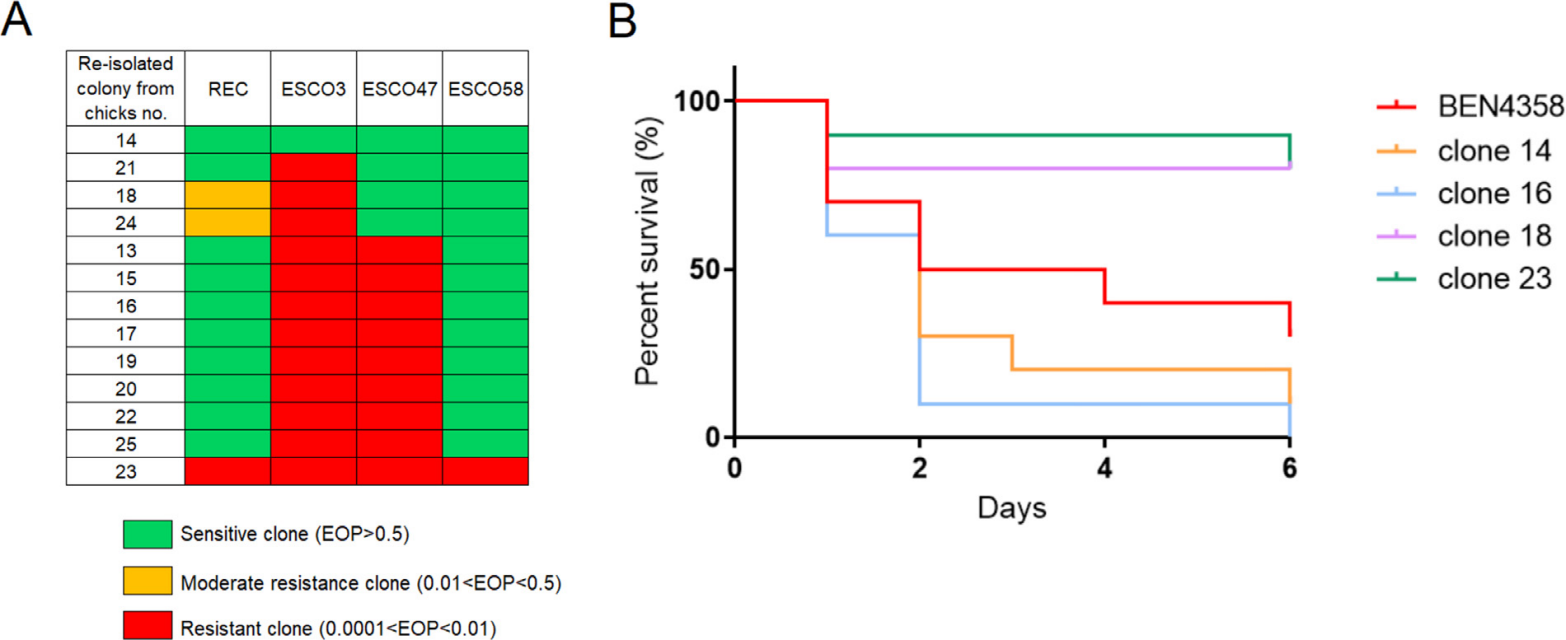
Phage resistance and impact on virulence of reisolated clones. (A) Efficiency of plating of REC, ESCO3, ESCO47, and ESCO58 phages on the reisolated colonies from the “EC + Phages” surviving chicks (*n* = 13), classified by resistance profile. EOP is the number of PFU obtained on a reisolated clone divided by the number of PFU obtained on the wild-type BEN4358 strain. (B) In ovo virulence testing of 4 reisolated resistant clones. Kaplan-Meier survival curves of embryonated chicken eggs. Eggs were inoculated with 239 CFU of *E. coli* BEN4358 strain (red curve), 281 CFU of clone 14 (orange curve), 274 CFU of clone 16 (blue curve), 205 CFU of clone 18 (pink curve), or with 234 CFU of clone 23 (green curve).

Phage sensitivity of 13 BEN4358 clones reisolated from the surviving chicks of “EC + Phages” group was determined (Figure 4A). One clone (no. 14) was sensitive to the 4 phages, like the wild-type BEN4358 strain. In contrast, 1 clone (no. 23) was fully resistant to the 4 phages. The other 11 exhibited intermediate phenotypes: clone no. 21 was only totally resistant to ESCO3; 2 clones (18 and 24) were totally resistant to ESCO3 and partially resistant to REC; 8 clones (13, 15, 16, 17, 19, 20, 22, and 25) were totally resistant to ESCO3 and ESCO47.

Phage resistance is frequently correlated with a cost of bacterial fitness, the virulence of clones 14, 16, 18, and 23 with 4 different resistance profiles was therefore evaluated in a chicken embryo lethality assay (Figure 4B). The phage-susceptible clone 14 and the ESCO3 and ESCO47 resistant clone 16 induced the death of 90 and 100% of embryos, respectively. There was no significant difference with the wild-type strain, which induced the death of 70% of the embryos (*P* > 0.05). In contrast, the ESCO3-resistant clone 18 and the fully resistant clone 23 were significantly (*P* = 0.03 and *P* = 0.02, respectively) less virulent than the wild-type strain, indeed only 20% of the embryos died. As the capsule and O antigen of lipopolysaccharide (**LPS**) are the virulence factors putatively targeted by phages for adsorption, their expression was determined. Capsular antigen K1 and O2 antigen were detected in all resistant clones (data not shown).

## DISCUSSION

Phage therapy is a promising alternative to antibiotic therapy, and several studies have reported its efficacy in the control of colibacillosis in poultry (Barrow et al., 1998; Huff et al., 2002a,b; Eid et al., 2022). Nevertheless, relatively few studies have administered phages by routes that are applicable in the field. In experimentally infected birds by APEC, phages administered via drinking water alone did not significantly reduce mortality and morbidity in experimentally infected birds (Huff et al., 2002b; Tsonos et al., 2014), and aerosol sprayed phages had an effect only when administered the same day as the infection (Huff et al., 2002a, 2003a). In hatcheries, vaccination by in ovo injection is commonly used. In addition, it has been shown that in ovo administration of innate immune stimulants (oligodeoxynucleotides and polyinosinic-polycytidylic acid) at ED18 protected 100% of the chicks against infection of the yolk sac by APEC O2:K1 (Sarfraz et al., 2022). To our knowledge, to date, in ovo administration of phages to prevent avian colibacillosis in chicks has never been performed. In the present study, phages were administered in ovo into the allantoic fluid at 17 d of embryogenesis. They were able to diffuse into the embryo body and were detected in 88% of newly hatched chicks. Among these phage-positive chicks, 64% had the 4 phages composing the cocktail. It was shown that a viral vaccine inoculated into the allantoic fluid at ED17 was found in only 3/12 newly hatched chicks, whereas when distributed into the amniotic fluid it was found in 100% of the chicks (Wakenell et al., 2002). Indeed, amniotic fluid is considered as the optimal site of in ovo distribution for many other products (hormones, probiotics, nutrients) (Peebles, 2018). Here, we show that the allantoic fluid is also an effective route for phage inoculation. However, to increase the number of phage-positive chicks at hatching, in future studies it would be interesting, to investigate the efficacy of delivering the cocktail into amniotic fluid at ED18.

Following the APEC infection of 1-day-old chicks, REC and ESCO3 phages multiplied in vivo since they were detected in surviving chicks at 7 dpi. In contrast, ESCO47 and ESCO58 phages were not detected, even though they were relatively stable in vitro and in ovo, and exhibited lytic activity against strain BEN4358 in the laboratory. But phage-bacteria interactions may differ between in vitro and in vivo conditions. For example, the phage receptor may be downregulated or not expressed in vivo. It has already been shown that bacterial receptors for lytic phages are downregulated in the murine gut, decreasing the sensitivity of an *E. coli* strain to phage infection (Lourenço et al., 2022). Phage-phage interactions should also be considered. Indeed, during coinfections, phages with a lower adsorption rate and latency will have more difficulty multiplying.

Birds were challenged with the APEC strain BEN4358 at 1 d of age. No difference in the mortality rate was observed between the groups. This could be explained by the insufficient concentration of phages at the time of challenge, since phages need to reach the threshold concentration to lyse a large number of bacteria (Payne and Jansen, 2003). Depending on the phage inoculated, this threshold may vary. Indeed, complete protection was achieved in chickens treated with 10^4^ PFU of phage R (Barrow et al., 1998), while only a reduction in mortality was observed when treated with 10^8^ PFU of phage SPR02 (Huff et al., 2006). Increasing the concentration of phages inoculated into the embryos could provide better protection. However, at 7 dpi, the surviving chicks that received the phages in ovo did not develop any lesions associated with colibacillosis, suggesting that the phages prevented the development of the infection. Similarly, it was shown that preventive treatment with SPR02 phage added to drinking water 1 wk prior to infection with an APEC O2 strain did not reduce chick mortality, but no lesions were observed in surviving chicks (Huff et al., 2002a). A significant decrease in the intestinal load of BEN4358 was observed in the present study. Few studies have analyzed the bacterial load in the intestinal reservoir following phage therapy in the context of colibacillosis, with the majority investigating instead the target organs of APEC strains (lungs, pericardium, and liver). It was shown that following oral treatment of phage ΦKAZ14, there was a significant 4-log reduction in the intestinal load of strain O1:K1 and an identical reduction in the strain excretion rate (Kaikabo et al., 2017). Thus, the 1-log reduction in the gut load of BEN4358 observed in our study, although slight, may contribute to reducing environmental spreading.

Despite constructing a cocktail of 4 phages belonging to distinct taxonomic genera and putatively targeting different receptors (Nicolas et al., 2023), phage-resistant variants were found in the ceca of surviving chicks. Phages and bacteria co-evolve, and the emergence of phage-resistant bacteria is a common phenomenon that could impact the success of phage therapy. The emergence of phage-resistant variants was observed in 82% of the studies that aimed to reduce intestinal colonization (Oechslin, 2018). Among the 13 clones derived from inoculated BEN4358 strain and recovered from the ceca of surviving chicks, 1 was sensitive to the 4 phages in the cocktail, like the BEN4358 strain, and 1 was fully resistant to the 4 phages. Twelve clones were fully resistant to ESCO3. However, this phage was still able to multiply in vivo, suggesting that a minority population of susceptible bacteria was maintained. Several mechanisms could explain this coexistence: a different spatial distribution between sensitive and resistant populations; the ability of the phage to evolve to counteract bacterial resistance; or the maintenance of a population of sensitive bacteria because of the selective disadvantage that phage resistance can bring (Hampton et al., 2020; Lourenço et al., 2020). In contrast, resistance to *Phapecoctavirus* REC was not as prevalent. It seems that this resistance is associated with a bacterial fitness cost, since among phage-resistant clones, only those which were completely or partially resistant to REC were less virulent than the wild-type strain. This alteration in virulence following the development of phage resistance is frequently reported in phage therapy trials (Oechslin, 2018; Mangalea and Duerkop, 2020). However, bacteria use many and varied strategies to resist to phages (Georjon and Bernheim, 2023) and further studies would be needed to identify the affected gene(s). The reduced virulence of REC-resistant clones would suggest that REC targets genes essential involved in virulence. As the K1 capsule and O2 antigen, which are known to be major virulence factors of APEC (Guabiraba and Schouler, 2015), were both detected in REC-resistant clones, others virulence factors are probably affected, as well as the modification of one or more nonvirulence genes being associated with fitness trade-off. Indeed, it was previously shown that an APEC O1 strain resistant to the T4-like phage P10 suffered a significant fitness cost following mutations in genes related to bacterial growth (acetate kinase) or in the beta-oxidation cycle of fatty acid degradation (Sørensen et al., 2021). Clearly, further studies of REC and BEN4358 phage-bacteria interactions would be required to identify the phage target and to improve our understanding of the dynamics during phage therapy. Furthermore, these results emphasize the therapeutic potential of *Phapecoctavirus* REC. Phages of this genus are easy to isolate since they are present in many ecosystems (human microbiota, avian microbiota, and wastewater) (Khalifeh et al., 2021). Also, *Phapecoctaviruses* are found in the Microgen ColiProteus cocktail, marketed in Russia, indicating a therapeutic potential against *E. coli* strains (McCallin et al., 2013).

In summary, we demonstrated that 4 phages inoculated into allantoic fluid of chicken embryos were transmitted to newly hatched chicks and protected them from developing avian colibacillosis when challenged. In addition, a 1-log decrease in the intestinal load of the APEC strain BEN4358 was observed, which may contribute to reducing environmental spread. Some clones evolved to develop varying degrees of phage resistance and depending on their resistance, some clones were less virulent than the wild-type strain. Like vaccines, the main challenge for phage therapy will be to provide complete protection against the multiple APEC strains circulating on farms. In future studies, it would be of interest to test the cocktail's efficacy by challenging the birds with other APEC strains and to test also different challenge routes reflecting more what happens in field conditions (oral and respiratory routes).
